# Emotional engagement and perceived empathy in live vs. automated psychological interviews

**DOI:** 10.1371/journal.pone.0323490

**Published:** 2025-05-21

**Authors:** Thomas J. Nyman, Anna-Karin Noromies, Francesco Pompedda, Pekka Santtila, Jan Antfolk

**Affiliations:** 1 School of Psychology and Clinical Language Sciences, University of Reading, Reading, United Kingdom; 2 Faculty of Arts, Psychology and Theology, Åbo Akademi University, Turku, Finland; 3 Faculty of Arts and Sciences, New York University Shanghai, Shanghai, People’s Republic of China; 4 Shanghai Frontiers Science Center of Artificial Intelligence and Deep Learning, New York University Shanghai, Shanghai, People’s Republic of China; 5 INVESThub, INVEST Research Flagship Centre, University of Turku, Turku, Finland; University of Lahore - Raiwind Road Campus: The University of Lahore, PAKISTAN

## Abstract

In clinical in-person conditions, social presence, perceived empathy, and emotional engagement are related to positive outcomes. In online settings, it is unclear how these factors affect outcomes. Here, in 10–15-minute interviews, we investigated the influence of automation. Participants (*N* = 75) engaged in one of three possible interviews: live semi-scripted, live scripted, or video scripted. In the first two, participants communicated with a live interviewer and, in the third, with pre-recorded interviewer questions and answers. Emotion recognition software revealed that expressed joy differed between conditions (*χ*^2^(2) = 18.08, *p* < .001); both live conditions had higher scores (vs. video scripted). Self-rated perceived interviewer empathy also differed between conditions in the same way (*F*[2, 72] = 9.445, *p* < 0.001). We found a positive correlation between perceived empathy and expressed joy (*r* = .35; *p* < .01). In sum, automatized interviews differed in perceived empathy and expressed emotion compared with live interviews.

## Introduction

According to the World Health Organization (WHO), globally one in eight individuals suffer from a mental disorder, with an estimated yearly cost of approximately $2.5 trillion in 2010, which is expected to rise to $6 trillion by 2030 [1]. The efficacies of psychological and psychotherapeutic treatments and interventions have been well established [2], nevertheless access is limited with less than 50%, and in some countries less than 10%, of individuals suffering from mental disorders receiving adequate treatment. The problem has been aggravated by the negative effects of increased waiting times [3,4] and, more recently, during the COVID-19 pandemic due to the global disruption in mental health services [5]. Finding new ways of reaching those in need of psychological treatment and offering evidence-based interventions is becoming increasingly important. Digital mental health interventions (DMHIs) present one clear path to providing such interventions [6]. The key components in successful psychological therapies include the therapeutic alliance and the emotional engagement of both the therapist and the client [7,8]. Consequently, research is still needed to understand how to successfully deliver online interventions and to what extent this process, at least in some cases, can be automatized [6].

The present study aimed to investigate how the automation of a human presence in a 10–15-minute online psychological interview on wellbeing affects factors that are central to the therapeutic alliance; that is, the emotional engagement and reactions of the interviewees and the empathy they perceived from the interviewer. To achieve this aim, we created three conditions ranging from a normal online interview (live semi-scripted), a more rigid interview (live scripted), and an automated condition (video scripted). In each interview condition, we used pre and post self-reports and objective emotion recognition software to measure the reactions of the interviewees and their perceptions of the interviewer.

### Digital mental health interventions

Internet-based technology offers ways to overcome some of the barriers that can hinder the possibility of receiving face-to-face treatment (e.g., physical travel distance to clinics or hospitals), which has prompted research on internet-based mental health interventions [9]. Overall, digital mental health interventions (DMHIs) have evolved over the past 25 years [6] and can offer several benefits relative to traditional face-to-face interventions. For example, they can be delivered through personal devices and potentially to any location at any time, which is crucial for those who do not currently have access to mental health treatment [10,11]. Some DMHIs (e.g., internet-based cognitive behavioral therapy) are presently as effective as traditional face-to-face interventions and many researchers and practitioners are advocating that they be researched and utilized to a greater degree [6,12–14].

To date, one of the most widely studied online methods is internet-based cognitive behavioral therapy (iCBT), which has been found to be as effective as face-to-face interventions for a variety of different psychiatric conditions (e.g., phobias and social anxiety), demonstrating both short-term and long-term effects [15]. This approach is based on cognitive behavioral therapy, an evidence-based treatment method that focuses on improving the way an individual feels by challenging unhelpful thoughts and behaviors, which can help them overcome psychological problems [16]. Research on internet-based interventions indicates that the effectiveness and adherence to internet-based interventions can be improved by human support [6,17].

Nevertheless, studies on unguided or automated internet-based interventions delivered by a computer or mobile phone with no human input or presence have also been found to be effective [18–22]. It has even been found that such methods can have long-term effects [23]. However, adherence to unguided interventions can often be low [17,24–28]. Attempts have been made to improve automated interventions by including a simulated human presence (i.e., a virtual representation of a psychologist) and this has been proven to foster a therapeutic relationship with the program [29–31]. For example, Pinto and colleagues [30,31] found that participants experienced a sense of rapport and social presence with the simulated avatar of a health-care professional. Their results show that the group who interacted with the avatar had significantly fewer depression symptoms compared to the attentional control group (receiving a computer-based health education). Pinto and colleagues [31] speculated that this could be related to the interaction with the avatar.

In traditional face-to-face therapies, the therapeutic relationship formed with the therapist predicts a positive treatment outcome [8] and it is likely that this is also an important factor in internet-based interventions. However, research on online methods, such as iCBT, indicates that online approaches may not always require live human support to be effective and that simulated human presence can be as effective as a traditional method. This highlights the need to further understand the role that human presence plays in online treatment processes and the minimum amount and form of presence necessary for a treatment to be effective. Indeed, while guided online interventions often result in better outcomes, and are favored by clients [32,33], they are less scalable, more expensive and more difficult to implement compared to unguided interventions [6,33,34]. Automated programs are an attractive alternative to traditional methods since they would be a cost-effective alternative offered to individuals that may otherwise be placed on a waiting list. Nevertheless, although internet-based interventions show promise as alternative methods for treating less complex psychological problems, online methods may not necessarily be suitable for more severe psychological problems [6,35].

### Social presence in clinical interactions

An essential aspect of the human-computer interaction is the subjective experience of contact with the real or artificial other, also referred to as social presence [36]. Social presence has been shown to contribute to positive communication outcomes in mediated environments [37], thus making it relevant to the design of computer programs in health care and technologies simulating clinical interactions [6,36]. A technological feature that influences the sense of social presence is the communication modality used [37]. It has, for example, been found that text-based computer-mediated communication (CMC) evokes less social presence than richer forms of media (e.g., video, audio, or avatar) [38]. Studies have, however, shown that with longer interactions even media with richer social cues can evoke an equally viable sense of social presence and contact that is comparable with to face-to-face interactions. This is because individuals adapt to less rich social cues and take on other communication strategies such as direct questioning or self-disclosure [39–41].

Another important influential factor on social presence is visual representation. Previous research has concluded that the extent to which a visual representation performs in a similar manner to a human being (i.e., behavioral realism) has positive effects on perceived social presence [38,42,43]. For example, Von Der Pütten and colleagues [43] found that participants felt a higher social presence when a computerized agent nodded its head compared to an agent that did not. Similar effects have also been found for maintenance of mutual eye contact [38], and a virtual agent blushing strongly after making a mistake during a presentation [42]. Researchers have also shown that the effect of behavioral realism is dependent on the appearance of the representation. For example, the more realistic a representation looks, the more realistic its behavior needs to be in order for a higher social presence to be evoked [44,45].

To date, there is no widely accepted, validated, and generalized measure of presence across varied media or settings, due to the different existing conceptualizations of presence [44]. Nevertheless, social presence has often been operationalized either in terms of an individual’s perceptions that another person is present or in terms of an individual’s social response to the other [44]. In the present study, we did not explicitly evaluate social presence but instead manipulated the social presence of the interviewer through an experimental design (live semi-scripted, live scripted, or video scripted). We then asked participants to self-rate their own emotional engagement and to rate the perceived empathy of the interviewer. We also measured the emotional reactions of the interviewees during the interactions.

### The therapeutic alliance, emotional engagement, and perceived empathy

It has been shown that a strong therapeutic alliance or relationship (i.e., the emotional bond and agreement concerning the tasks and goals of the treatment between the client and the therapist) is an important predictor of positive outcomes in traditional face-to-face psychotherapy and counselling [8]. A central element of the therapeutic alliance is the emotional engagement of both the therapist and the client, and it has also been shown that the empathic ability of the therapist is a predictor of a client’s progress [7,8]. Moreover, the perception of a therapist as empathetic contributes to the development of a positive therapeutic alliance [46,47] and promotes therapeutic change by facilitating client initiative, social interaction, and engagement [47].

Interestingly, it is specifically the perceived empathy by clients or patient rather than by observers or therapists that has been shown to be a medium-sized predictor of therapeutic outcomes [7]. This highlights the importance of the perceiver’s experience in face-to-face therapy. However, empathy is a complex concept [48] and knowing how and when (and when not) to explicitly display empathetic behavior in an interaction is a skill that denotes sensitivity to the individual, the context, and the working relationship [7]. Moreover, a therapeutic process does not only entail displays of empathy but may also include instances of challenging the thoughts and judgements of a client or patient [49], which further illustrating that how to act as a therapist requires skill. Lastly, considering that empathy is more of a co-production between therapist and client or patient rather than something solely displayed by a therapist [7], it is also important to consider that there are metaperceptual aspects to judging what another is thinking. Here, findings show that the metaperception of an individual concerning another’s thoughts of them can be a substantial source of error [50]. Therefore, perceived empathy may also be associated with differences in the ability of an individual, such as a client or patient, to evaluate the empathy displayed by another, such as a therapist. Overall, there is still much research needed to understand the role of perceived empathy in therapeutic contexts.

In studies on guided internet-based interventions, it has been found that the working alliance (i.e., the agreement on tasks and goals of the treatment) has been rated to be as positive and as stable as that in face-to-face interactions [51–53]. However, in internet-based interventions the working alliance can rarely be considered to be a predictor of a positive outcome [15,54]. For example, Knaevelsrud and Maercker [51] found that the alliance in a guided iCBT program for posttraumatic stress reactions was not as clearly related to the outcome compared to face-to-face approaches, despite high ratings of a therapeutic alliance. It has also been found that a client can develop a relationship with a computer program [55–58]. This relationship is possible because individuals tend to treat computers as social beings, a tendency that is known as the Computers as Social Actors (CASA) paradigm, which implies that an individual reacts to the social cues provided by a computer, an avatar, or an algorithm [59–61]. While people attribute social responses to computers, little is known whether computer interactions are perceived as empathetic and emotionally engaging. As computer interactions are becoming more frequently used in clinical interactions, knowledge about how they are perceived and experienced is required due to their increasing significance in clinical interactions.

### The current study and hypotheses

The current study had two main aims: First, in 10–15-minute online psychological interviews on wellbeing, we investigated whether the pre- and post-measures of valence and activation, emotional reactions, and perceptions of empathy differed when participants interacted with a live semi-scripted online interviewer (live semi-scripted condition) versus an automated interviewer (live video scripted condition). In the live semi-scripted condition, the participants were seated in a laboratory in front of a computer and communicated online with a live interviewer. Here, the interviewer used a semi-scripted approach to ask and answer questions in the online interview. In the live video scripted condition, the participants were also seated in a laboratory in front of a computer, but here they were communicating with pre-recorded video clips and not a live human being. They were not informed that they were interacting with pre-recorded video clips. Instead, we simulated a live online discussion by presenting participants with a seamless integration of pre-recorded video clips of questions and answers. The pre-recorded questions and answers were recordings of the same interviewer as in the live semi-scripted condition. During the live video scripted condition, the test instructor (not visible to the participant) listened to what the responses of the participant and selected, in accordance with a decision-logic, what pre-recorded question or answer clips to present to the participant. In this way, we simulated an automatized online discussion and participants were led to believe that they were conversing with a real human being.

Second, we wanted to compare the live semi-scripted condition to a live scripted condition. In the live scripted condition, the participants were seated in a laboratory in front of a computer and communicated online with a live interviewer. However, here the interviewer was not as free to choose answers as in the semi-scripted condition. Instead, the live scripted condition followed the same decision-logic as the video scripted condition. We added the live scripted condition to evaluate the difference between the decision-logic and the video presentation. Consequently, if the live semi-scripted gave rise to different emotional reactions or engagement or perceived empathy, we wanted to understand if that was due to the format (i.e., the pre-recordings) or the script (i.e., the decision-logic). Our setup resulted in three computer-based online interview conditions: 1) live semi-scripted, 2) live scripted, 3) video scripted.

Furthermore, in accordance with the CASA paradigm [59], we assumed that the interviewee would interact with the automated interviewer (i.e., video scripted condition) as if communicating with a live interviewer. Thus, we expected that the video scripted condition would elicit similar reactions and emotional engagement in the participant as in the live conditions, consistent with studies on social reactions to computers and avatars [43,62]. This was also because the interviews were short and highly consistent. However, we also assumed that the live interactions would contain richer social cues compared to the automated interaction (i.e., video scripted condition) and could thus evoke a higher social presence. The rigidity of the interaction in the video scripted condition might negatively affect the feeling of social presence, as the interaction would be less spontaneous and natural. Overall, we expect the conditions to evoke similar responses, however, if the rigidity was found to lead to a decreased perception of social presence or engagement, we should be able to detect differences between by comparing all three conditions. Additionally, we wanted to investigate whether self-rated emotional engagement, emotional reactions, and perceptions of interviewer empathy correlated positively. This was in part because previous research has shown that perceived empathy contributes to an overall positive alliance [46,47]. Our hypotheses were that the increased automation of presence (i.e., less rich social cues) would lead to the following:

Hypothesis 1 (H1): Lower self-rated emotional engagement by the interviewees.

Hypothesis 2 (H2): Lower positive emotional reactions from interviewees.

Hypothesis 3 (H3): Lower ratings of perceived empathy of the interviewer.

To summarize, in all three hypotheses (H1-H3), we expected the live semi-scripted condition to evoke the highest ratings of perceived emotional engagement, emotional reactions, and perceptions of empathy, followed by lower ratings in the live scripted condition, and the lowest ratings in the video scripted condition. However, based on studies on social reactions to computers and avatars [43,62], we also included an alternative consideration – that we might not detect differences between conditions. This might be the case if the video scripted condition would elicit similar reactions and emotional engagement in the participant as in the live conditions. Additionally, we also hypothesized that:

Hypothesis 4 (H4): There will be a positive correlation between engagement, reactions, and perceived empathy.

## Methods

### Participants

The participants were contacted through mailing lists at universities and vocational schools, through social media, and by recruitment in the university canteen. Our sample consists of 75 Swedish-speaking students recruited at Åbo Akademi University. The recruitment and testing took place from 25/10/2017–20/03/2018. The sample consists of 49 female participants (*M*_age_ = 22.67, *SD* = 2.31) and 25 male participants (*M*_age_ = 24.68, *SD* = 6.12). One participant did not report age or gender. The age of the participants ranged from 19 to 52 years (*M*_age_ = 23.35, *SD* = 4.09; *n* = 74). The participants were pseudo-randomized into three separate experimental conditions with 25 participants per condition: 1) the live semi-scripted group (*M*_age_ = 24.04, *SD* = 6.17, female = 19, male = 6), 2) the live scripted group (*M*_age_ = 23.24, *SD* = 2.77, female = 17, male = 8), and 3) the video scripted group (*M*_age_ = 22.75, *SD* = 2.11, female = 13, male = 11). Participants received a lunch coupon for their participation.

### Ethics statement

All participants were informed of the voluntary nature of the studies, their right to end their participation at any stage (without giving a reason), and a written and fully informed consent was obtained from all participants. The current study was approved by the Research Ethics Committee of Psychology and Logopedics at Åbo Akademi University.

### Design

The experimental setup was a between-subjects design with three conditions: 1) live semi-scripted, 2) live scripted, and 3) video scripted. In the live semi-scripted condition, participants were seated in a laboratory and interviewed live via a computer by a psychologist graduate student who followed a pre-defined and fixed order list of questions and answers (see Procedure). In this condition, the interviewer could respond by freely validating or paraphrasing the interviewee’s responses either non-verbally or verbally before continuing to the next question. The interview was conducted as a video call (including both audio and video). In the live scripted condition, participants were also seated in a laboratory and interviewed live via a computer by a psychologist graduate student. Here, during the video call the interviewer was verbally limited to six pre-defined answers and used a decision-logic to decide which answer to give (see Procedure). Lastly, in the video scripted condition, participants were seated in a laboratory and interviewed live via a computer by pre-recorded video clips that were seamlessly presented on the computer screen to mimic a live video call interview. Each pre-recorded video clip was played on the screen by the test instructor who was listening to (but could not see) the participants responses. The video clips to be played (i.e., the pre-recordings of the interviewer questions and answers) followed the same order as the live semi-scripted and scripted condition. Additionally, the presentation of the video clips was limited to the same six pre-defined interviewer answers as in the live scripted condition. The test instructor used the same decision-logic to decide which response to play as was used in the live scripted condition. The participants were not informed that they were interacting with pre-recorded video clips in the video scripted condition but were instead informed that they would participate in a live video call interview for all conditions. The test instructor was the same person as the interviewer and all interviews (live and video) included the same interviewer.

The independent variable was the automation of presence (i.e., richness of social cues) and was represented by the degree to which the answers followed a decision-logic that was lenient (live semi-scripted) or rigid (live scripted and video scripted) and to what extent the interview was conducted by a live human (live scripted and live scripted) or represented by pre-recorded video clips (video scripted). The dependent variables were the participant self-ratings of emotional engagement, emotional expressions during the experiment, and post-interview ratings of the degree to which the interviewer was empathic.

### Materials

We used an iMac desktop (24-inch, 1920x1200, early 2009) and a Hewlett Packard desktop (HP Compac 8200 Elite MT PC) to conduct the interviews in all three conditions. To conduct the live interviews, we used the website *Doxy.me* (© Doxy.me, LLC 2014). *Doxy.me* was chosen since it was considered to comply with HIPAA (the Health Insurance Portability and Accountability Act) and HITECH (the Health Information Technology for Economic and Clinical Health). HIPAA is a standard for privacy and security rules that protect patient data.

For the video interview, we filmed the interview questions and responses using a digital camera (Canon EOS 1300D; lens: Canon EF 50mm f/1.8). The video recordings were recorded to capture the psychology student from shoulder-height upwards and were edited with *OpenShot Video Editor*. In total, the recorded material consisted of 27 video clips, which included four clips of the introductory part of the interview, 14 clips of the main interview questions, one clip that functioned as a bridge when changing the theme in the interview (i.e., to produce a seamless viewing experience when presenting different clips), one clip that was the concluding part of the interview, six clips representing the different interviewer responses, and one clip that functioned as a dynamic background clip. To simulate the live scripted interview, we further edited the video-clips and then displayed them using *Resolume Arena 5* so that the video clips could transition seamlessly during the interview. In the *Resolume Arena 5* software tool we created a composition of video clips organized in layers and in the desired order. The test instructor operated this simulation remotely in the video scripted condition.

### Measures

#### Symptoms of depression.

We used a Swedish translation of the Patient Health Questionnaire (PHQ-9) to investigate the intensity of symptoms of depression during the previous two weeks. We included this measure because depression has been shown to reduce emotional responses [63]. We chose the PHQ-9 measure since it is suitable as a screening tool for depression in research settings and shows good validity and reliability [64]. The answers were scored on a four-point Likert-type scale ranging from 0 (not at all) to 3 (almost every day), with a total score ranging from 0 to 27. Scores ranging from 0–4 are categorized as minimal, 5–9 as mild, 10–14 as moderate, 15–19 as moderately severe, and 20–27 as severe [64]. In addition, we also collected the following demographic data: gender, age, as well as previous experience of counselling or psychotherapy. The last of which was collected because experience of interaction with a mental health professional could affect the expectations of the participants and thus also affect the perceptions of and reactions of the participant toward the interviewer. The demographic data were obtained via a background questionnaire.

#### The self-rated emotional engagement of the interviewees.

To assess the emotional engagement of the participants (H1), we measured self- reported emotional engagement and facial expressions. We assessed the perceived emotional state of the participants using the self-report measure Swedish Core and Affect Scale (SCAS), administered before and after the interview. This tool was used because all interviews were conducted in Swedish. The SCAS measures the valence and activation of the current mood of the individual based on an affect grid [65]. The 12 pre and 12 post items are graded on a 9-point Likert-type scale ranging from (-) 4 to (+) 4, with the end points of the scale defined by adjective pairs (e. g., sad-glad, bored-interested, engaged-disengaged). In the SCAS measure, participants are asked to rate how they felt at that very moment (at both pre and post interview) regarding the following items: 1) displeased–pleased 2) sad–glad, 3) depressed–happy, 4) sleepy–awake, 5) dull–peppy, 6) passive–active, 7) bored–interested, 8) indifferent–engaged, 9) pessimistic–optimistic, 10) tense–serene, 11) anxious–calm, 12) nervous–relaxed. Moreover, the SCAS measure allows for the creation of a composite of valence and activation. The pre- and post-valance composite and the pre- and post-activation composite were calculated after imputing the missing data for these variables (see *Statistical Analyses*) based on the average of three variables for valence (pleased, glad, and happy) and the average of three variables for activation (awake, peppy and active). This calculation was based on the original works [65,66]. The SCAS measure shows adequate reliability and validity for composite scores of valence and activation [66].

#### The emotional reactions of the interviewees.

We measured the emotional reactions of the participants in the interviews with automated facial expression analysis (H2). Facial expression analysis provides a simple, low time consuming, and less intrusive measurement of emotion and shows adequate sensitivity to the valence of emotional states [67–69]. This analysis was performed with the use of the *AFFDEX* Software Development Kit (SDK) 2.0 [70]. Using the built-in computer camera, the program software analyzes facial action units (AU) (i.e., facial movements) and classifies specific combinations of these units as emotions, based on a facial coding scheme [71]. In a validation study of the *AFFDEX* software it was found (Study 1) that the overall accuracy of valence was 73% for picture-based prototypical facial expressions and 55% (study 2) for video-based dynamic facial expressions [72]. The emotions were coded on a scale from 0 to + 100. We calculated the mean value for all the seven emotions measured by the *AFFDEX* software development kit *(*SDK). The emotions measured were anger, sadness, disgust, joy, surprise, fear, and contempt.

#### The perceived empathy of the interviewer.

We measured the reactions of the participants to the interviewer and the participant’s perceptions of the interviewer (H3) using a Swedish translation of the Consultation and Relational Empathy (CARE) measure [73]. The CARE measure is considered to have high acceptability, face validity, and internal construct validity [74] and is designed to evaluate the extent to which the patient judges the consultant to be empathic in a one-on-one consultation. The CARE measure includes 10 questions that are graded on a six-point scale ranging from 1 (poor) to 5 (excellent), including a Not Applicable (NA) option [75]. Additionally, the ten questions are combined to produce a composite score of empathy, which ranges from 10–50. The ten questions are based on asking how good was the doctor at: 1) Making you feel at ease, 2) Letting you tell your “story”, 3) Really listening, 4) Being interested in you as a whole person, 5) Fully understanding your concerns, 6) Showing care and compassion, 7) Being positive, 8) Explaining things clearly, 9) Helping you take control, 10) Making a plan of action with you.

### Procedure

Before conducting the experiment, we piloted the experimental design in a small sample (*n* = 10) of university students who were not included in the main study. We found no previously validated structured clinical interview for our experiment, so we devised a draft version and tested the interview to establish its comprehensibility and functionality, and the functionality of the pre-recorded video interview. No alterations were made between the pilot and the main experiment. None of the participants reported any disturbing or irritating aspects of the pre-recorded video interview, however, for the live interviews some reported being disturbed by occasional desynchronization of audio and graphics, which was likely due to a poor internet connection.

We conducted the interviews in rooms on the university campus. We asked the participants to take part in an approximately 10–15-minute online interview concerning their health and everyday life (i.e., wellbeing). At this point they were given incomplete information regarding the nature of the study, and we did not reveal the specific hypothesis concerning the effect of the automation of presence (i.e., less rich social cues) on emotional engagement and perceived empathy. This was necessary for us to test our hypothesis in a valid manner. The participants were assured anonymity and were informed that participation was voluntary and could be discontinued at any time. Upon arrival, a research assistant welcomed and gave each participant instructions. The setup was a double-blind as the assistant was unaware of the research question, the study design, and the group to which the participant had been assigned. The participants read, agreed, and signed a standard consent form before starting the experiment.

Once seated in the test room, the participant first completed a background information questionnaire, then the Patient Health Questionnaire (PHQ-9) and finally the Swedish Core and Affect Scale (SCAS). The participant was then repositioned in front of an iMac computer at an adjoining table, asked to put on headphones, and wait for the interview to begin. We used the *Doxy.me* platform and the *AFFDEX* software program on the iMac. Participants also used sound-dampening headphones to hinder them hearing the interviewer talk in the adjacent test room (in addition to hearing them via the headphones via the computer) – that is, the participants were led to believe that the online interviewer was communicating from a separate location, while the interviewer was in fact in the adjacent room.

**Fig 1:**
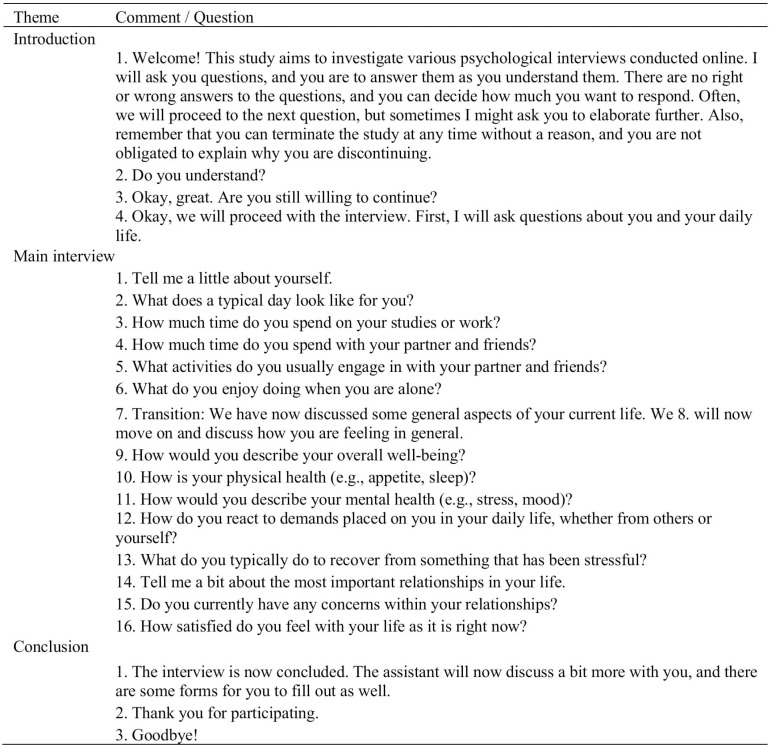
The interview structure, comments, and questions. The figure represents the order of questions and comments presented in the psychological interview for all experimental conditions. The theme refers to the stage of the interview and the comment/question refers to the comments and questions asked by the interviewer. In the live conditions the questions were asked in the live video call, whereas in the video scripted condition, the comments/questions were presented as pre-recorded video clips of the interviewer commenting/asking. The original interview language was Swedish, and the comments and questions in Fig 1 have been translated to English by the first author (a native English speaker) for this publication.

Prior to the interview, the research assistant also instructed the participants to avoid changing their sitting position and to avoid turning their head during the interview. The reason for this was to enable the *AFFDEX* software to identify facial expressions and analyze them as accurately as possible during the interview. In each condition, the interview followed the same interview structure, thus allowing for comparisons between groups (see Fig 1).

**Fig 2:**
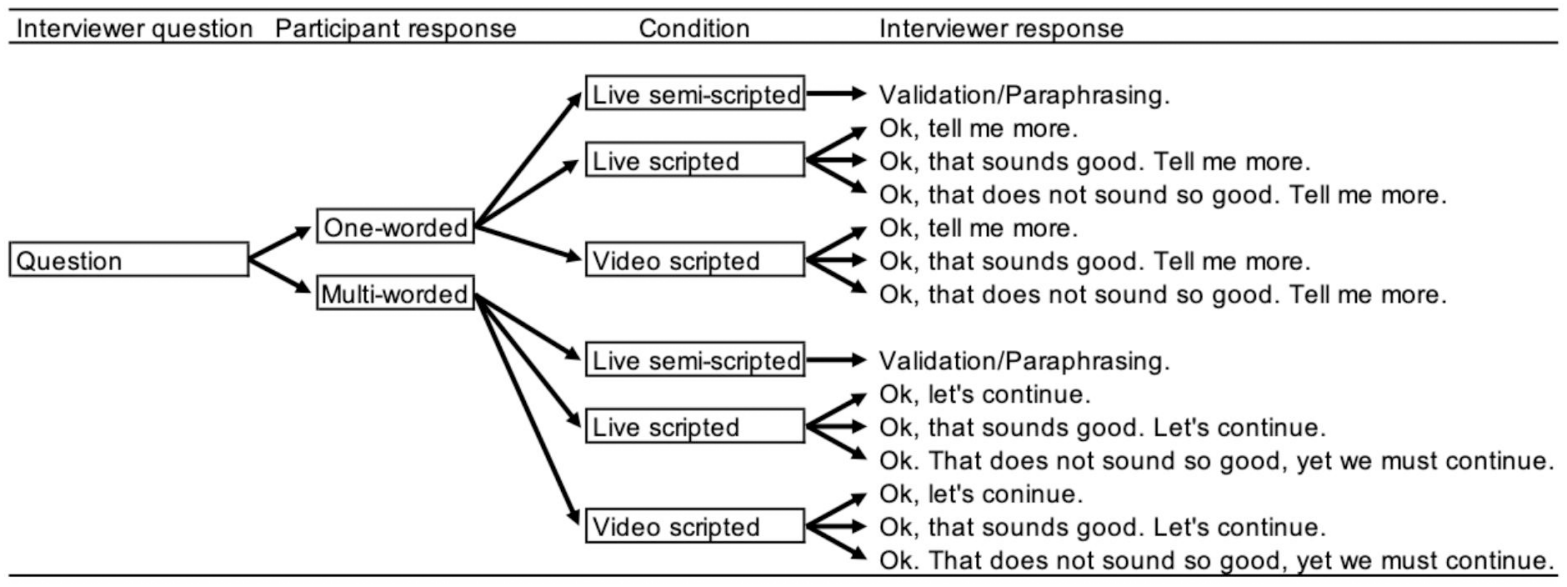
The decision-logic of the interviewer during the psychological interview. Schematic of the response decision-logic. In the live semi-scripted interview, each response of the participant was followed by a validating paraphrasing statement. In the live scripted and video scripted interview, each response of the participant was followed by either a line trying to engage the participant or a line indicating that the interview should continue to the next question. The original interview language was Swedish, and the interviewer responses in Fig 2 have been translated to English by the first author (a native English speaker) for this publication.

The introductory part of the interview was to establish contact, explain the aims of the interview, and restate that their participation could be discontinued at any time without explanation, and obtaining a verbal affirmation to continue. The main part of the interview consisted of open questions (e.g., “Tell me about a typical day in your life”, “How would you describe your overall health?”) relating to central themes discussed in an intake interview [76]. In the live semi-scripted condition, the interviewer was to a limited extent free to choose how to respond to the participant’s answer. We operationalized the term “free” as spontaneously being able to validate or paraphrase what the participant has said (e.g., “You say that...”, “It seems like you think that…”, “If I understand you correctly you say that…”). The live scripted and the video scripted conditions differed in the number of ways the interviewer could respond. In these two conditions, the interviewer’s answer followed a decision-logic based on the response of the participant (see Fig 2).

During the post-interview, the participant completed the SCAS questionnaire and the Consultation and Relational Empathy (CARE) questionnaire. Immediately after completion, the research assistant thanked them for their participation and gave the study participants a debriefing document. The debriefing document contained information concerning the real purpose of the study (however, the hypotheses were not disclosed), relevant background information pertaining to the study, the contact information of the researcher for follow-up questions and the study results, and information about counselling services in the city.

### Statistical analyses

All analyses reported in this article were conducted using R [77] and the specific packages and their references have been specified for each analysis.

#### Missing data of the self-rated emotional engagement of the interviewees.

We evaluated all variables in the dataset for missing data. In the pre-test SCAS answers, which contained 12 questions, we found a total of four missing values from four participants (i.e., 0.004%) which were all in the live scripted condition. Additionally, we found 10 missing values all from one person in the post-test SCAS answers (i.e., 0.011%). The pattern of missing values for the pre-test SCAS answers suggests that they are missing at random (MAR) [78,79]. However, for the post-test SCAS answers, the patterns of missing data were due to only one participant: hence not missing at random (NMAR). We have addressed the missing values by using the R package “mice” [80], which is a Bayesian multiple imputation method that employs Markov Chain Monte Carlo (MCMC) algorithms. This is considered a robust approach and recommend to be used with MAR data [81]. Here, we also calculated the missing values within each condition and not across the entire dataset to better represent the responses within each condition and any differences there may be between conditions. For the NMAR data, we adopted two strategies: 1) we used the same approach to the imputation as for the MAR data, 2) we ran the analysis twice, once with the participant present with imputed data and once with the participant excluded. As there were no differences between the two analyses, we have reported results with the participants included.

#### Missing data of the emotional reactions of the interviewees.

Subsequently, when evaluating the *AFFDEX* data we found a total of 49 missing observations from seven participants (five participants in the live scripted group, two in the live group, and no participants in pre-recorded group). The data loss was a result of computer software failure. The *AFFDEX* software stopped in cases where the program was started before signing into the website *Doxy.me*. Moreover, as we only used *Doxy.me* in the live semi-scripted and live scripted interviews, this software failure was only possible in these conditions, thus related to the group variable. As there was no other clear pattern, we have treated this as MAR. For this reason, we once again employed the R package “mice” to impute the missing values. Lastly, we did not exclude any outliers in the *AFFDEX* dataset because any extreme values could represent an aspect of the inherent variability of the data (i.e., outliers can be legitimate emotional reactions in an online setting).

#### Missing data of the perceived empathy of the interviewer.

In the CARE questionnaire we received 128 Not Applicable (NA) responses. The majority of the NA responses (99) were given for questions 9 (*Helping you to take control*) and 10 (*Making a plan of action with you*). These NA answers are not specifically missing because they represent answers where the participants did not feel that the questions applied. As there was no other clear pattern, we have treated this as MAR and again used the Bayesian multiple imputation method to deal with the NA responses. Lastly, due to the large number of participants in all three conditions who felt that questions 9 and 10 were not applicable, we have interpreted these results with caution.

#### Analysis of the self-rated emotional engagement of the interviewees.

Our first hypothesis (H1) was that the increased automation of the interviewer would decrease the emotional engagement. This meant that we expected the self-rated emotional engagement of the interviewees to be the highest in the live semi-scripted condition followed by lower ratings in the live scripted condition, and the lowest ratings in the pre-recorded video condition. To investigate H1, we performed a mixed-model ANOVA with time as the within-subject factor and condition as the between-subject factor. We used the valance composite and activation composite of the SCAS measure as dependent variables. To investigate the main and interactive effects, we used the afex package [82]. To investigate Tukey post hoc comparisons, we used the emmeans package [83].

#### Analysis of the emotional reactions of the interviewees.

Similar to H1, our second hypothesis (H2) was that increasing the automation of the interviewer would decrease the emotional reactions among the interviewers. To investigate if the total amount of expressed emotional reactions differed between groups and if there were differences between groups in emotions, we aimed to conduct a one-way MANOVA on all *AFFDEX* data, followed by one-way ANOVAs on each emotion separately. The dependent variables were the average of each of the seven measured emotions. For the MANOVA we used the car package [84] and for the multivariate normality tests we used the MVN package [85]. However, due to multiple assumption violations, we replaced the MANOVA analysis by running multiple non-parametric Kruskal–Wallis tests (with Bonferroni corrections). Additionally, to further investigate the main and interactive effects, we conducted non-parametric pairwise multiple comparison tests using the Dunn test via the dunn.test package [86].

#### Analysis of the perceived empathy of the interviewer.

Our third hypothesis (H3) was in line with H1 and H2. Here, we expected that the automation of the interviewer would decrease the perceived empathy of the interviewer. To investigate differences between groups on perceived empathy, we performed a one-way ANOVA with the condition as the predictor and the composite empathy (based on the CARE measure) as the outcome. Moreover, due to violations of normality we also followed up with a Kruskal–Wallis test. To investigate the main and interactive effects, we conducted post-hoc Tukey tests. Following this, we aimed to run a MANOVA of the ten CARE measures, but, instead, due to multiple assumption violations we conducted multiple Kruskal–Wallis tests (with Bonferroni corrections), followed by post-hoc Dunn tests.

#### Analysis of the measures of engagement, reactions, and perceived empathy.

To investigate our fourth hypothesis (H4), which was that self-rated engagement, emotional reactions, and perceptions of empathy of the interviewer would be correlated, we conducted correlational analyses between the pre and post SCAS measures, the AFFDEX emotion measures, and the CARE empathy measure. Due to earlier assumption violations, we opted for the non-parametric Spearman’s correlation.

## Results

### Balance checks of the experimental conditions

Prior to the main analyses, we confirmed that each condition had twenty-five subjects. Next, we investigated whether there were any group differences based on the PHQ-9 scores, gender, and previous experience. When investigating group differences based on the PHQ-9 scores, we found that the assumption of normality was violated (Shapiro-Wilk test, *W* = 0.92192, *p* < .001). Based on a substitute non-parametric Kruskal–Wallis test, we found no evidence of significant differences in median scores across groups (*χ*^2^(2) = 0.691, *p* = 0.708). We then investigated group differences based on gender, by running a Chi-Square test and found that gender was equally distributed across groups (*χ*^2^(2, N = 74) = 2.663, *p* = .264). Lastly, using a Chi-Square test, we found that previous experience of psychotherapy or counselling did not differ significantly between groups (*χ*^2^(2, N = 74) = 0.239, *p* = .888). Accordingly, the variables PhQ-9, gender, and previous experience of psychotherapy or counselling were excluded from subsequent analyses.

### The self-rated emotional engagement of the interviewees

We aimed to conduct a mixed-model ANOVA to assess the effects of condition (live semi-scripted, live scripted, or video scripted), SCAS composites (valence or activation), and time (pre or post) on participants’ emotional engagement measured as the SCAS composite score. Assessing the assumptions of data distribution, we found that both normality (Shapiro Wilk, *W* = 0.993, *p* = .204) and homogeneity of variances (*F*[11, 288] = 1.34, *p* = .202) were upheld. Subsequently, we conducted a mixed-model ANOVA and found no main effects of condition (*F*[2, 72] = 0.65, *p* = .526) or the SCAS composites (*F*[1, 72) = 2.59, *p* = .112), but we did find a main effect of time (*F*[1, 72] = 37.63, *p* < .001). Additionally, there was a significant interaction between the SCAS composites and time (*F*[1, 71] = 14.70, *p* < .001). No other interactions were significant.

For the interaction effect between SCAS composite and time, Tukey post hoc comparisons showed that participants’ valence composites post-interview (*M* = 1.54, *SD *= 1.51) were significantly higher compared to the valence composites pre-interview (*M* = 1.15, *SD* = 1.41, E = 0.40, *SE* = 0.12, 95% CI [0.08, 0.71], *p* = .008). Similarly, the activation composites of participants post-interview (*M* = 1.61, *SD* = 1.37) were significantly higher compared to the activation composites pre-interview (*M* = 0.60, *SD* = 1.91, *E* = 1.00, *SE* = 0.16, 95% CI [0.59, 1.43], *p* < .0001). Moreover, as the post-SCAS measures included NMAR data from one participant, we also re-ran the analysis after excluding this one participant (#38). The exclusion of the participant did not change the main results, so we have only reported the results that include the participant.

The results indicate that the live semi-scripted, the live scripted, and the video scripted conditions elicited similar emotional engagement among our participants, with valence and activation increasing between the pre and post measures. The fact that valence and activation increased signifies that participants were engaged by the experimental setup.

### The emotional reactions of the interviewees

To evaluate group differences in emotional reactivity during the interview, we first conducted a MANOVA on the *AFFDEX* measures of facial expressions of emotions. Assessing the assumptions of the data distribution, we found that multivariate normality (Mardia’s test, *χ*^2^ = 875.08, *p* < .001) was violated and that the univariate normality assumption was violated for all individual emotional reactions (Anderson-Darling tests, for all *p* < .001). Additionally, when evaluating assumptions of homogeneity of variance, we found that contempt (Bartlett’s test *p* = .481) and surprise (*p* = .514) satisfied the criterion, while the other emotional reactions displayed violations (all *p* < .01). Lastly, we found that sphericity was violated (Mauchly’s Test, *W* = 1.4482e-06, *p* < .001). Therefore, to address the multiple assumption violations, we used non-parametric Kruskal–Wallis tests with Bonferroni corrections to evaluate the group differences of expressed facial emotions.

The results only revealed a significant difference for expressed joy (*χ*^2^(2) = 18.08, *p* < .001). Follow-up post-hoc analyses using the Dunn test revealed no significant difference in expressed joy between the live semi-scripted (*M* = 15.00, *SD* = 16.11) and live scripted (*M* = 18.28, *SD* = 19.99) conditions (*Z* = 0.63, *p* = .265). However, there were significant differences between the live semi-scripted and video scripted (*M* = 4.24, *SD* = 6.52) conditions (*Z* = 3.36, *p* < .001) and between the live scripted and video scripted conditions (*Z* = 3.98, *p* < .001).

The findings suggest that the live semi-scripted, live scripted, and video scripted conditions elicited overall similar emotional reactions, except for expressed joy. Notably, expressed joy was markedly higher in both the live semi-scripted and live scripted compared to the video scripted condition.

### The perceived empathy of the interviewer

To investigate group differences of the effect of the automation of presence (i.e., less rich social cues) on the interviewees’ perceptions of the interviewer, we first performed a one-way ANOVA on the group differences for composite empathy (i.e., composite score of perceived empathy). Assessing the assumptions of the data distribution, we found that normality was violated (Shapiro-Wilk test, *W* = 0.96018, *p* = .019) but the homogeneity of variance was upheld (Levene’s test, *F*[2, 72] = 0.338, *p* = .714). The results from the one-way ANOVA revealed a significant main effect of condition on empathy (*F*[2, 72] = 9.445, p < 0.001). Due to the violation of normality, we also conducted a Kruskal–Wallis test and confirmed the main effect of condition on empathy ratings (*χ*^2^(2) = 16.934, *p* < .001). A Tukey post-hoc test revealed no difference between the live semi-scripted (*M* = 38.87, *SD* = 8.27) and the live scripted (*M* = 38.53, *SD* = 6.24) conditions (*E* = 1.34, *SE* = 2.08, 95% CI [-3.62, 6.31], *p* = .795). However, there was a significant difference between the live semi-scripted and the video scripted (*M* = 31.47, *SD* = 7.37) conditions (*E* = 8.40, *SE* = 2.08, 95% CI [3.43, 13.37], *p* < .001) and between the live scripted and the video scripted conditions (*E* = 7.06, *SE* = 2.08, 95% CI [2.09, 12.02], *p* = .003).

Next, we aimed to conduct a MANOVA, but when assessing the assumptions of the data distribution we found that the homogeneity of variance (Shapiro-Wilk test, *W* = 0.96018, *p* = .019) and sphericity (Mauchly test, *W* = 0.002, p < .001) were violated. To address the multiple assumption violations, we employed Kruskal–Wallis tests with Bonferroni correction to evaluate group differences for the CARE measures.

We found significant differences in four of the ten measures. Specifically, we found differences between conditions for the CARE variables 1 (making you feel at ease; *χ*^2^(2) = 17.305, *p* < .001), 5 (fully understanding your concerns; *χ*^2^(2) = 16.055, *p* < .001), 6 (showing care and compassion; *χ*^2^(2) 12.504, *p* = .001), and 7 (being positive; *χ*^2^(2) = 16.577, *p* < .001). We ran follow-up post-hoc analyses (Holm-Bonferroni corrected) using the Dunn test for each of these four CARE measures.

The post-hoc analyses of the CARE 1 measure (making you feel at ease) revealed that there was no significant difference between the live semi-scripted (*M* = 4.28, *SD* = 0.89) and the live scripted (*M* = 4.02, *SD* = 0.77) conditions (*Z* = -1.13, *p* = .129). However, there were differences between the live semi-scripted and video scripted (*M* = 3.08, *SD* = 1.08) conditions (*Z* = 4.03, *p* < .001) and between the live scripted and the video scripted conditions (*M* = 3.08, *SD* = 1.08) (*Z* = 2.90, *p* < .001).

The post-hoc analyses of the CARE 5 measure (fully understanding your concerns) demonstrated that there was no significant difference between the live semi-scripted (*M* = 3.92, *SD* = 1.15) and live scripted (*M* = 3.54, *SD* = 1.08) conditions (*Z* = -1.12, *p* = .131). However, there were differences between the live semi-scripted and video scripted (*M* = 2.55, *SD* = 1.19) conditions (*Z* = 3.89, *p* < .001). and between the live scripted and the video scripted conditions (*Z* = 2.77, *p* = .003).

The post-hoc analyses of the CARE 6 measure (showing care and compassion) revealed no significant difference between the live semi-scripted (*M* = 4.04, *SD* = 1.17) and live scripted (*M* = 3.72, *SD* = 0.98) conditions (*Z* = -1.25, *p* = .105). However, there were significant differences between the live semi-scripted and video scripted (*M* = 2.76, *SD* = 1.31) conditions (*Z* = 3.49, *p* < .001) and between the live scripted and video scripted conditions (*Z* = 2.24, *p* = .013).

The post-hoc analyses of the CARE 7 measure (being positive) showed that there was no significant difference between the live semi-scripted (*M* = 4.28, *SD* = 0.89) and live scripted (*M* = 4.36, *SD* = 0.81) conditions (*Z* = 0.25, *p* = .403). However, there were significant differences between the live semi-scripted and video scripted (*M* = 3.26, *SD* = 1.03) conditions (*Z* = 3.40, *p* < .001) and between the live scripted and video scripted conditions (*Z* = 3.64, *p* < .001).

Overall, similar to the composite CARE empathy score, the investigation of individual CARE measures demonstrated that the live semi-scripted and the live scripted did not differ but both conditions differed markedly from the video scripted. The results suggest that our video scripted condition had a negative effect on perceived empathy, especially for four measures: 1 (making you feel at ease), 5 (fully understanding your concerns), 6 (showing CARE and compassion), and 7 (being positive).

### The correlation between engagement, emotional reactions, and perceived empathy

**Fig 3:**
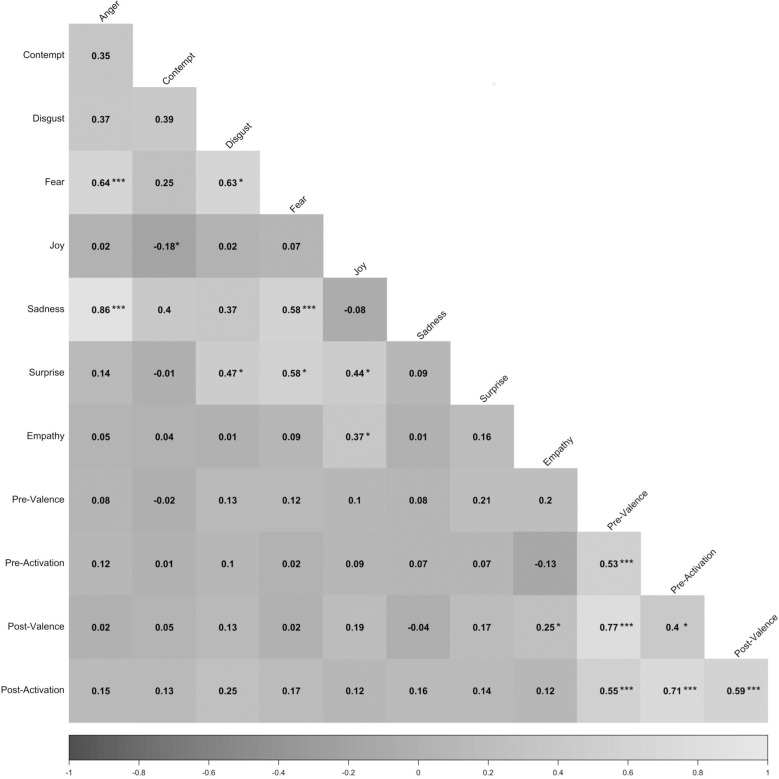
Correlation table of the pre- and post-measures. The matrix values represent Spearman’s rank correlations (*r*), and the significant correlations are represented as follows: * = *p* < .05, ** = *p* < .001, *** = *p* < .0001.

Lastly, we investigated the correlation between emotional engagement (SCAS: pre/post valence and activation), emotional reactions (*AFFDEX*: facial emotion expressions) and perceived empathy (CARE: empathy composite). See Fig 3 for the correlation table.

The most interesting correlations were those between perceived empathy and expressed joy (*r* = .35; *p* < .01). While causality cannot be inferred based on these analyses, the results suggest that participants may have had a more positive experience (i.e., greater positive change in affect and expressed more joy) when they perceived the interviewer as empathic.

Additionally, we found that several emotions were correlated. For example, we found a negative correlation between joy and contempt (*r* = -.20; *p* < .05), which is not surprising considering that joy is a positive emotion and contempt is a negative emotion. Moreover, we found correlations between fear and anger (*r* = .65; *p* < .0001), fear and disgust (*r* = .62; *p* < .05). and between fear and sadness (*r* = .59; *p* < .001). These correlations could be a result of the poor ability of the *AFFDEX* software to correctly identify fear and anger [72], implicating that some of the other emotions (e.g., sadness) could have been mistaken for fear. Other positive correlations between emotions on the *AFFDEX* measures were also found; these correlations suggest that participants who expressed one emotion were likely to express other emotions.

## Discussion

In our experiment the aim was to test how the automation of social presence (i.e., less rich social cues) would affect the experience of the interviewees in an online 10–15-minute psychological interview on wellbeing. We included three conditions: 1) a live semi-scripted online interview, 2) a live scripted online interview, and 3) a video scripted online interview. The three conditions represented our operationalization of degrees of automation of social presence. We were interested in evaluating how participants would react to these conditions and, therefore, we measured their pre and post self-reported emotional engagement (SCAS), their emotional engagement during the interview (*AFFDEX*), and their perceived empathy of the interviewer post interview (CARE).

Based on the background literature on human-computer interactions that richer social cues increase social presence [37], we anticipated that the increased automation of presence (i.e., less rich social cues) would decrease emotional engagement, reactions and perceived empathy. This would result in perceived emotional engagement, emotional reactions, and perceived empathy receiving the highest scores in the live semi-scripted condition, followed by the live scripted condition and last the video scripted condition. However, we also anticipated that the video scripted condition could elicit similar reactions and emotional engagement in the participant as in the live conditions, based on studies on social reactions to computers and avatars [43,62]. Therefore, our alternative consideration was that we might not detect differences between conditions. Lastly, we also analyzed the relationship between self-rated emotional engagement, emotional reactions, and perceptions of empathy. Here, we anticipated that there would be a positive correlation between self-rated emotional engagement, emotional reactions, and perceptions of empathy, since perceived empathy has shown to contribute to a positive alliance, and thus also the emotional engagement in the interaction [46,47].

Based on a sample of 75 participants (25 per condition) we were able to investigate our hypotheses and have outlined our main findings and interpretations below.

### The self-rated emotional engagement of the interviewees

The outcome of our analysis of emotional engagement indicated that automation had no significant effect on either activation or valence, but there was an interaction between time and our SCAS composite measures (pre and post activation and valence). Post-interview valence (*M* = 1.54, *SD* = 1.51) was higher compared to pre-interview valence (*M* = 1.15, *SD* = 1.41). Similarly, post-interview activation (*M* = 1.61, *SD* = 1.37) was higher compared to pre-interview activation (*M* = 0.60, *SD* = 1.91). The results indicate that valence and activation increased between our pre to post measures, which signifies that participants were engaged by the experimental setup. Interestingly, there is an increasing amount of research showing that people tend to treat computers as social beings and react to them as if they were humans [59,60], and that people respond socially even more strongly to computer characters such as avatars due to their more pronounced human-like attributes [43]. The lack of significant differences between the conditions on activation and valence could be related to how the interaction was perceived. The pre-recorded video interaction might have led interviewees to believe that the interaction was a live human. Perceiving the interaction as human-operated influences the social presence positively while perceiving the interaction as computer-operated negatively affects social presence [87]. This could potentially mean that a real-life human psychologist interviewer could be replaced by an automated system of pre-recorded videos of questions and answers – at least in some instances.

### The emotional reactions of the interviewees

When analyzing the facial expression of emotional reactions, we found that emotional reactions were similar in all conditions except for expressed joy, which was higher in both the live semi-scripted (*M* = 15.00, *SD* = 16.11) and live scripted (*M* = 18.28, *SD* = 19.99) compared to the video scripted condition (*M* = 4.24, *SD* = 6.52). The facial expression measure is perhaps a more objective measure (vs. self-reports) of the interviewee’s emotional reactions as it is a non-intrusive and real-time measure of a participant’s emotional reactions. The results provide some support for our hypothesis that the increased automation of presence decreases emotional reactions. A possible explanation for the difference in joyful expressions but not on other emotions is that *AFFDEX* detects happy expressions more often. *AFFDEX* identifies dynamic happy expressions particularly well (91% of the time), compared to the accuracy for other dynamic expressions: anger (49%), contempt (68%), disgust (79%), fear (1%), sadness (35%) and surprise (61%) [72]. The reason for this could be that happiness is the most distinctly expressed emotion, whereas fear and surprise are more often confused (since they are characterized by similar markers). In line with the results of the self-reported emotional engagement, the outcome of the emotional reactions in the pre-recorded video condition also supports the existing research that people react socially to computers and human representations [59].

### The perceived empathy of the interviewer

The automation of presence was found to influence perceive empathy (CARE composite), so that the live semi-scripted (*M* = 38.87, *SD* = 8.27) and the live scripted (*M* = 38.53, *SD* = 6.24) both had significantly higher ratings compared with the video scripted condition (*M* = 31.47, *SD* = 7.37). Additionally, examining the individual CARE measures, we found that the live conditions had higher ratings for four measures: 1 (making you feel at ease), 5 (fully understanding your concerns), 6 (showing CARE and compassion), and 7 (being positive). In line with our expectations, interviewees experienced the interviewer as most empathic in the live conditions and least empathic in the video scripted condition.

The outcomes suggest that the conditions differ in richness of social cues. This resonates with previous research showing that, for at least shorter interactions, media with richer social cues evoke more social presence than media with less rich social cues [38,44]. A possible explanation for the lower empathy reports could be that the pre-recorded video interview was perceived as less realistic than the live interviews. Previous studies have shown that social presence is experienced when behavioral realism is higher [38,42,43] and that incongruity between photographic and behavioral realism yields lesser experience of social presence [37]. Another possibility for the lower empathy reports in the video scripted interview is that the pre-recorded video interview was perceived as controlled by an algorithm. Perceiving the interview as controlled by a computer has been shown to negatively impact social presence compared to interviews that are perceived as being controlled by a human [88]. However, as we did not directly ask participants about their perceptions of realism of the interviews, this is speculation (see also limitations). Future studies could measure perceived agency to discount such explanations.

Lastly, in contrast to our expectation that the rigidity in the live scripted condition would lead to lower ratings of perceived empathy compared to the live semi-scripted condition, we found no significant difference between these conditions. Previous studies have shown that consistency between the level of realism of the visual appearance and the level of realism of the behavior increases social presence [44,45]. We argue that this result suggests that the live-scripted condition, where the flexibility of the verbal responses was restricted, was perhaps not perceived as less realistic than the live condition. Increased rigidity through adherence to a script might not have led to a significant decrease in richness of social cues, by which significant differences between the live and live-scripted condition could be detected. This is perhaps because social cues such as adequate eye contact, smiling, head nods and vocal pitch were still present in both conditions. Overall, our findings provide partial support for our hypothesis that increasing the automation of presence (i.e., less rich social cues) decreases perceived empathy, and suggests that a live interaction differs from a pre-recorded video interaction regarding perceptions of empathy.

### The correlation between engagement, emotional reactions, and perceived empathy

We investigated the relationship between self-rated emotional engagement, emotional reactions, and perceived empathy. Our hypothesis was that there would be a positive correlation between the measures. In line with our hypothesis, participants who perceived the interviewer as more empathic were found to more likely have a positive experience (i.e., greater positive change in activation of affect and more expressed joy), which might indicate that perceiving the interviewer as more empathetic contributed to a more positive experience. This outcome mirrors the research on the importance of empathy for the development of positive alliance [46,47]. Our results suggest that the video scripted interaction was perceived and experienced differently (i.e., as less empathetic and eliciting fewer joyful expressions) compared to a live interaction with a human. This indicates that empathy has an impact on how online psychological interviews are experienced and that automation may reduce aspects that are central to positive psychological outcomes.

### Limitations

There are a few limitations that are important to mention. First, in the video scripted condition, the test instructor could not see the interviewee on the computer screen while operating the interview simulation, and was, therefore, only able to hear the participant. For this reason, we cannot be certain that the participants had their head turned to the camera during the whole interview. If so, the *AFFDEX* software would not be able to analyze facial expressions since it is not applicable in different head poses. However, the *AFFDEX* data does suggest that the participants did have their heads turned to the camera as the program makes interpretations every second. Second, in each condition once or twice the *AFFDEX* data was missing for 20–60 seconds during the interview for a few participants. Third, as was discussed in the results section, we encountered missing values in the *AFFDEX* data in the live and live-scripted condition due to a software malfunction. Fourth, we encountered bandwidth delays at times in the live interviews, which was likely due to a poor internet connection. However, it should be added that bandwidth delays are an inherent aspect of video conference communication and is as such a reflection of actual online communication.

Fifth, there was sometimes a lack of synchronized audio and graphics in the video scripted interview, and this could potentially have influenced how realistic the interview appeared and be a potential confounding variable. However, synchronization issues might also have been interpreted as bandwidth issues, which would reduce that potential confound. Sixth, the accuracies of the AFFDEX measures are not without their limitations and, therefore, due to the errors rates of the measures, these results need to be treated with some caution when generalizing. Nevertheless, we argue that evaluated together with the other self-reports these results present a balanced evaluation of our manipulations.

Lastly, in the present study we did not explicitly measure social presence and there are therefore some limitations to the generalizability of our results. We manipulated the social presence of the interviewer by creating conditions that represented various degrees of automation. In so doing, we did achieve different types of social presence, but as our measures concerned emotional engagement and perceived empathy, we could only evaluate social presence indirectly. Moreover, as we did not explicitly ask participants if they were aware at any point that the interviewer was automated (in the fully automated condition) it is unclear to what extent they perceived it to be realistic and how that perception may have impacted their engagement.

## Conclusions

In conclusion, our results add to the field of research aiming to examine the role of human presence in online psychological interventions and to the field of human-machine communication. As automated programs are emerging in clinical settings and people are now interacting with a computer instead of a human, it is interesting that little is known about how automated interactions are perceived and experienced. A deeper understanding of the role of human presence is needed for the development of automated internet-based self-help programs. It is also important to find ways to enhance the use of these programs by making them more engaging, since to date, adherence to these programs is low. To our knowledge, this is the first study investigating whether there are differences between a live semi-scripted online interaction, a live scripted interaction, and a video scripted interaction regarding social presence.

Our results suggest that the automatization of an interviewer decreased the perceived empathy of the interviewer and decreased the emotional engagement of the interviewees in terms of expressed joy; however, it did not affect the self-rated engagement of the interviewees. Furthermore, increased rigidity through adherence to a script, in the live scripted condition, did not significantly impact perceived empathy, emotional reactions, or expressed joy, compared with the live semi-scripted condition. Instead, the negative effect of automation was only found in the video scripted condition. However, although we were able to manipulate social presence experimentally, we did not explicitly measure social presence but instead measured it indirectly through perceived empathy and emotional engagement. Moreover, given that the lack of consensus of how to define the concept of social presence, our results may be difficult to compare to other studies on social presence. A key feature that was not addressed in the present study was how the perception of the other as a real human or how realistic the scenario was impacted our measures – this may be an important avenue for future research. Nevertheless, we have attempted to contribute to the ongoing discussion of how to understand social presence, perceived empathy, and emotional engagement in online psychological interventions and the field of human-machine communication.

Considering the significant technological advancements since this study was conducted, future research should replicate these findings to investigate if psychological interviews can be automatized and still retain the beneficial elements of live interactions.
